# Friend or Foe? An Unrecognized Role of Uric Acid in Cancer Development and the Potential Anticancer Effects of Uric Acid-lowering Drugs

**DOI:** 10.7150/jca.46200

**Published:** 2020-07-06

**Authors:** Shuyi Mi, Liang Gong, Ziqi Sui

**Affiliations:** 1 Department of Gastroenterology, The First People's Hospital of Yuhang District, Hangzhou, Zhejiang Province, China.; 2 Department of Otolaryngology, Cixi People's Hospital, Ningbo, Zhejiang Province, China.; 3 School of Medicine, Zhejiang University City College, Hangzhou, Zhejiang Province, China.; 4 Department of Pathophysiology, College of Basic Medical Sciences, Jiamusi University, Jiamusi, Heilongjiang Province, China.

**Keywords:** hyperuricemia, cancer risk, metabolic syndrome, liver metastasis, uric acid-lowering agents

## Abstract

In recent years, metabolic syndrome (Mets) has been a hot topic among medical scientists. Mets has an intimate relationship with the incidence and development of various cancers. As a contributory factor of Mets, hyperuricemia actually plays an inseparable role in the formation of various metabolic disorders. Although uric acid is classically considered an antioxidant with beneficial effects, mounting evidence indicates that a high serum uric acid (SUA) level may serve as a pro-oxidant to generate inflammatory reactions and oxidative stress. In this review, we describe the unrecognized role of hyperuricemia in cancer development and summarize major mechanisms linking uric acid to carcinogenesis. Furthermore, we also discuss the potential mechanism of liver metastasis of cancer and list some types of uric acid-lowering agents, which may exert anticancer effects.

## Introduction

In recent years, metabolic syndrome (Mets) has been a hot topic among medical scientists. Mets indicates a cluster of metabolic abnormalities including abdominal adiposity, insulin resistance, hyperglycemia, hypertension, and dyslipidemia, causing an increased morbidity and social economic burdens 1. Increasing evidence has shown that Mets has a close relationship with the incidence and development of some specific cancers, such as breast cancer, ovarian cancer, and pancreatic cancer 2-4. However, as a contributory factor of Mets, hyperuricemia plays an important role in the formation of various metabolic disorders, including diabetes, obesity, hypertension, and so on 5, 6. While it is assumed that Mets and cancer share common underlying mechanisms of oxidative stress and inflammation 4, we have good reason to believe that hyperuricemia is also detrimental to cells, which has not been widely studied in the occurrence and development of cancer.

Hyperuricemia, or increased serum uric acid (SUA) level, is defined as an average SUA level > 6.8 mg/dL (404 µM) with or without the recognized complication of gout 7. With a dual role, uric acid is the end product of purine metabolism via xanthine oxidoreductase, which is mainly eliminated by the kidney and the intestinal tract 8. When uricemia is in the normal range, with its powerful antioxidative activity, uric acid is thought to scavenge free radicals and contribute to the total antioxidative capacity of plasma 9. Studies have indicated that uric acid provides a protective role in red blood cells and nerve cells by providing an antioxidant defense 10, 11. It is also assumed that the radical scavenging action of circulating uric acid decreases neoplastic transformation, to reduce the risk of cancer 4. However, a study has recently reported that the antioxidant activity of uric acid is not as strong as either hydrophilic vitamin C or hydrophobic vitamin E, and is unlikely to play an important protective role by quenching oxygen radicals 12. In addition, serum uric acid in very high concentrations may trigger inflammatory stress, and it may also have intracellular pro-oxidative activity 4, 13. It is well-known that a pro-oxidant environment confers a growth advantage to tumor cells, and also influences carcinogenic potential by stimulating specific signaling cascades that regulate cell growth and apoptosis 14.

An increasing number of recent studies have reported a positive correlation between certain types of cancer and Mets, but as the central entity linking Mets to inflammation and cancer, uric acid has been neglected, and studies of the relationship between SUA levels and cancers have been limited. In this review, therefore, we describe the relatively unrecognized role of high serum uric acid in cancer development and metastasis and discuss some of the major mechanisms linking uric acid to carcinogenesis. Because high SUA levels might promote the development of cancer, it is assumed that uric acid-lowering agents are able to exert some benefits in the treatment of cancer. With the increasing resistance to traditional cancer drugs, we believe the use of uric acid-lowering therapy may constitute a novel strategy for the management of some refractory cancers.

## Uric acid as a risk factor in various cancers

Hyperuricemia is the consequence of purine metabolism disorders, which can lead to tumorigenesis and cancer 15. Increasing numbers of studies have recently suggested that a high level of SUA is associated with higher cancer incidence and mortality 16, 17. In addition, it was reported that there exists a site bias of digestive organs and urological organs in the relationship between high SUA levels and cancer mortality 15, 18.

A statistically significant association was found between higher SUA levels and increased mortality of total cancers, especially the specific sites of digestive cancer, which is also more significant in females than males 18. After eliminating preclinical diseases, a Chinese study suggested that elevated SUA was independently and positively connected with the risk of digestive cancer and cancer mortality among hypertensive Chinese 19. Also, a study using mouse model without gene of urate oxidase, has observed that the majority of mice spontaneously developed hepatocellular carcinoma by the age of 2 years 20, which indicates the potential role of hyperuricemia in cancer development. For nasopharyngeal cancer, low post-treatment plasma uric acid levels may be better for patients who benefit from additional aggressive treatment after intensity-modulated radiotherapy, implicating low SUA as possibly conducive to cancer therapy 21. Evidence has shown that preoperative SUA is an independent prognostic predictor in esophageal squamous cell carcinoma patients who undergo R0 esophagectomy, and patients with a higher SUA level might have significantly shorter 1, 3, and 5 year survival times than patients with a relatively low SUA level 22. For colorectal cancer (CRC), it was found that SUA levels gradually increased from stage I to stage IV, suggesting that the SUA level reflected the severity of CRC and may help to evaluate the therapy effect as well as the prognoses of CRC patients 14. In addition, an elevated serum level of uric acid was shown to be a significant prognostic marker for lymphatic metastasis in patients with colon cancer 23. Among pancreatic cancer patients, it was also observed in a large cohort study that elevated uric acid levels were an independently poor prognostic factor for overall survival 24.

In addition to its association with the development of digestive cancer, SUA levels also correlate with the incidence of urological cancers. It was found that gout patients had a higher risk of prostate cancer, followed by bladder and renal cancers 25. A Swedish study of males with metabolic syndrome showed that high SUA levels were an independent significant predictor of prostate cancer 26. It was reported that a postoperative increase of ≥ 10% in the SUA level was predictive of decreased overall survival (OS) and recurrence free survival (RFS) in stage I-III renal cell carcinoma patients, while improved OS and RFS were observed in patients with decreased/stable SUA levels at both 5 and 10 years 27.

It is also not rare for cancer patients, especially patients with lymphocytic leukemia and Burkitt's lymphoma, to have a high SUA level, among whom a high plasma uric acid level may occur as a result of increased purine metabolism by xanthine oxidase as a consequence of tumor cell breakdown 28. A high SUA level was associated with a poor prognosis in acute myelocytic leukemia patients 29. It was demonstrated that diffuse, large B-cell lymphoma patients having elevated SUA levels showed consistently worse conditional survival outcomes when compared with patients with lower uric acid levels, with their conditional outcomes only approaching those of the lower uric acid patients approximately 5 years after diagnosis 30. As for respiratory organs, it has been found that among the non-small cell lung cancer patients who had higher SUA levels; there was a higher percentage of brain metastasis, and a shorter time until brain metastasis and lower overall survival 31. In addition to leading to increased mortality and lower prognosis of cancer patients, hyperuricemia may also reduce the effectiveness of anticancer agents. It has been reported that the elevated SUA levels in hyperuricemia mice also negatively impacted on the effectiveness of immunotherapy to delay growth of melanoma 32.

Gout, defined as a progressive metabolic disease characterized by symptomatic hyperuricemia, is also considered a risk factor for cancer 33, 34. Additional evidence has shown that gout increases the risk of cancer, and a higher incidence from all causes of cancer has been found in the high prevalence of various gout-related comorbidities 15, 35. A nationwide population study investigating the relationship between gout and cancer found that the annual incidence of cancer in gout patients was more than double that of the normal population 36. In another study, SUA > 0.56 mmol/L and crystal-proven gout were found to be strongly associated with mortality and other chronic diseases 37. Moreover, it was found that a genetically determined lifelong high exposure to urate is more detrimental than high plasma urate, which develops later in life, because lifelong high plasma urate results in a higher risk of cancer and all-cause mortality 16.

## The main mechanisms of uric acid during cancer development: Inflammation and oxidative stress

There is increasing evidence supporting the potential role of uric acid metabolism in carcinogenesis, involving uric acid-induced inflammation and the production of reactive oxygen species (ROS) in the interaction between uric acid and the immune system 38-40. And it is well known that long-term chronic inflammation in tumor microenvironments has a great impact on neoplasia and tumor progression as well as on immunity, which also indicates the role of uric acid-induced inflammation in cancer development 41, 42.

When patients develop hyperuricemia, uric acid saturates body fluids and undergoes a phase change by nucleating into crystals of monosodium urate (MSU), which are the prime components that trigger symptoms and cause diseases 40. After its release from dying cells, uric acid is believed to be one of the damage-associated molecular patterns, which alert the immune system to an abnormal situation 43. As a result, uric acid is detected and leukocytes such as neutrophils and macrophages infiltrate tissues; dendritic cells can also be stimulated, causing acute inflammation 44, 45. When MSU particles are ingested by phagocytes, they stimulate the NOD-like receptor family, pyrin domain-containing 3 (NLRP3) inflammasomes to activate the caspase-1 protease, which cleaves IL-1β into its active form, thus eventually producing the proinflammatory form of the IL-1β cytokine 46.

The results of another recent study provide evidence that soluble uric acid (sUA) is also responsible for increasing IL-1β production in an Nlrp3- and Myd88-dependent manner 39. When cells from healthy subjects were pretreated with uric acid, it specifically downregulated the production of the anti-inflammatory cytokine IL-1 receptor antagonist (IL-1Ra) and, as a result, Toll-like receptor-induced proinflammatory cytokine production was significantly increased and that IL-1β is a signaling molecule secreted when the NLRP3 inflammasome is activated 47. Furthermore, some studies reported that urate-induced immune programming also plays an important role in immune injury 48. It is supposed that the upregulation of mRNA levels of inflammasome-related genes like IL-1β and NLRP3 are dependent on sUA production, which indicates sUA is able to alter the transcriptional program of the cell and modulate cytokine production, ultimately leading to the exacerbation of inflammation responses 49. Also, sUA gives rise to elevated serum chemokine (C-C motif) ligand 2 (CCL2) which is a chemoattractant recruiting circulating monocytes which play a part in chronic low-grade inflammation 50. Recently a study also observed elevated lipopolysaccharide and tumor necrosis factor-α in hyperuricemia mice, suggesting hyperuricemia mice were in low systemic inflammation 51.

Apart from inflammatory stress, it is also generally thought that ROS induced by uric acid play a central role in carcinogenesis 52. On the one hand, hyperuricemia mostly results from excessive activity of xanthine oxidase in catalyzing uric acid production, among which also exists excessive ROS production 8. On the other, it is supposed that uric acid exerts a pro-oxidant activity mainly in an intracellular way. NLRP3 inflammasome stimulation by sUA is accompanied by cellular redox state changes increasing in the mitochondrial area, resulting in increased mitochondrial ROS production 39. Meanwhile, uric acid crystals activate the immune system, acting as a pro-oxidant molecule that reduces nitric oxide availability, to increase the production of intracellular ROS 53.

The link between ROS and cancer promotion has been known for many years. It is thought that ROS play a dual role in the regulation of the tumor cell signaling pathway 54. ROS activate signaling pathways related to proliferation, survival, angiogenesis and metastasis, and promote the occurrence, development, and metastasis of tumors. Alternatively, a high level of ROS can induce cell apoptosis, promoting cell aging, and inhibiting the cell cycle 55. The potential effects of ROS on oncogenesis include: i) elevated ROS can activate Jun N-terminal kinase (JNK) and p38 mitogen activated-protein kinase (MAPK) signaling, which downregulates cyclins and induces cyclin-dependent kinase (CDK) inhibitors, resulting in cell cycle arrest 56. ii) Levels of ROS are correlated with the activity of matrix metalloproteinases (MMPs), and increasing MMPs in the tumor microenvironment induce tumor oncogenesis 57, 58. iii) ROS induce endothelial cell tube formation and the production of angiogenic factors, such as vascular endothelial growth factor (VEGF) and nitric oxide, which eventually accelerate angiogenesis 59. iv) ROS activate multiple pathways of the MAPK family to activate receptor tyrosine kinases (RPTKs) to promote the epithelial-mesenchymal transition (EMT) and also to create a premetastatic niche in distal organs, establishing a supportive environment for disseminated cancer cells 60. v) ROS can also activate integrin, induce focal adhesion kinase (FAK) phosphorylation, and promote tumor cell adhesion at the site of metastasis 61. vi) ROS imbalance leads to protein and lipid oxidation, increased mitochondrial membrane permeability, as well as changes of the coupling efficiency of the electron transfer chain, which produces more free radicals and cytochrome C, and activates apoptotic proteases and c-JNK, leading to cell apoptosis 62.

## Uric acid-lowering therapy and its ability to lower cancer risk

Surgical excision is still not effective for most cancers, so chemotherapy is still one of the important comprehensive treatments of cancers. However, with the application of dozens of anticancer or auxiliary anticancer drugs for clinical use, an increasing number of patients become therapeutically resistant. Considering that long-term, costly research is required to develop new drugs, drug repurpose is gradually becoming a novel strategy to treat cancer. The existing drugs like statin and aspirin, which are used to treat cardiovascular diseases, have now received a lot of attention for cancer therapy 63, 64. Because we now know the potential role of uric acid in cancer initiation and progression, there may also be uric acid-lowering drugs, which have the potential for treating cancer. It has been reported that metabolic remodeling plays an important role in the evolution of some cancers 65, so targeting different mechanisms in carcinogenesis using uric acid is consistent with the findings that uric acid lowering drugs are able to exert anticancer effects.

Microtubules are one of the components of the cytoskeleton, which play an important role in supporting cellular structure. In cancer chemotherapy, drugs that disrupt microtubule dynamics are used widely. Microtubules have long been considered an ideal target for anticancer drugs because of their essential roles in mitosis and formation of the dynamic spindle apparatus 66. Colchicine, known as an agent used to ameliorate acute gout attacks because of its microtubule disruption activities, has been studied for its possible anticancer effects 67. By targeting the colchicine-binding site on β-tubulin, colchicine inhibits microtubule polymerization and leads to prolonged metaphase arrest, thus playing an anti-cancer role as a microtubule inhibitor 68. In addition, protein expression of rearrangement during transection (RET) was found to correlate with larger tumor size, higher tumor stage, and decreased metastasis-free survival, while colchicine has recently been reported to decrease RET expression by selectively binding RET G-quadruplexes-DNA, which suggests a new mechanism for its anticancer activity 69. A study has also shown that colchicine induces cell death by apoptosis, and inhibits the invasion and migration of cancer cells by reducing extracellular matrix (ECM) degradation through downregulating MMP9 and the urokinase-type activator system 70. Actually, in some studies, colchicine in the form of nanoparticles has been used as an anticancer drug to inhibit colon and liver cancer cells 71, 72.

Other commonly used uric acid-lowering agents are xanthine oxidase inhibitors, such as allopurinol and febuxostat. It is known that xanthine oxidase catalyzes the formation of uric acid, as well as the production of ROS 73. The xanthine oxidase inhibitors are capable of blocking this pathway to reduce the production of uric acid and oxidative stress, which also involves its anticancer activity 74. It was shown that long-term (> 1 year) use of allopurinol resulted in a 34%-36% decrease in the risk of developing prostate cancer 75. Moreover, allopurinol was reported to attenuate ROS-induced signaling of cytokines, proteolytic activity, and tissue degradation in a rat model of cancer cachexia, which indicated its ability to reduce tissue wasting and improve survival from cancer cachexia 76. When combined with the tumor necrosis factor-related apoptosis-inducing ligand, allopurinol also strongly induced apoptosis in human hormone-refractory prostate cancer cells 77.

The liver is a frequent site of metastasis for various cancers, and non-alcoholic fatty liver disease (NAFLD) may be a significant factor in the liver microenvironment of cancer metastasis 78, 79. And uric acid regulates hepatic steatosis and insulin resistance through NLRP3 inflammasomes, playing an important role in the development of NAFLD 80. While it was also shown that allopurinol reduced uric acid and oxidative stress, therefore decreasing NLRP3 activation and IL-1β levels 81. Considering its role in attenuating NAFLD, allopurinol may also protect against cancer metastasis to the liver. It has been confirmed that allopurinol had the ability to reduce blood glucose levels and the induction of ROS, and alleviate hepatic oxidative stress, inflammation and steatosis, thus attenuating NAFLD 82. In the short-term fructose fed rat model, allopurinol prevented hepatic lipid peroxidation, protein oxidation, and acute adenosine triphosphate (ATP) depletion 83.

As for febuxostat, in addition to its ability to lower levels of serum uric acid, it also has a high affinity for endothelial-bound xanthine oxidase and, therefore, can reduce vascular ROS production, indicating that it may have a strong anticancer potential 84. Actually, because of its satisfactory efficacy in cancer cell lines, in the study using febuxostat nanoparticles as anticancer drugs for the treatment of lung cancer has suggested it as hopeful strategies 85. Compared with allopurinol, febuxostat decreased hepatic uric acid levels and xanthine oxidase activity in the non-alcoholic steatohepatitis (NASH) mouse model, which was accompanied by more effective prevention of certain features of NASH, including insulin resistance, lipid peroxidation, and liver inflammation, indicating greater efficiency in preventing liver metastasis 86.

## Discussion

In this review, we summarized current knowledge regarding the involvement of hyperuricemia in promoting the progression of various cancers and reducing cancer-related overall survival, which suggested that uric acid might be a potential risk factor of cancer development. Inflammation induced by both sUA and urate crystals as well as ROS production during the interaction between uric acid and immune system are thought to constitute the underlying mechanism stimulating the growth of cancer cells. In the presence of high SUA levels, cancer metastasis is also more likely to occur, which is possibly the consequence of alterations of the organ microenvironment before cancer metastases 23, 31.

The liver is a frequent site of metastasis for various cancers because of its unique biological characteristics 78. To some extent, hyperuricemia may promote the hepatic metastasis of cancer by affecting the liver microenvironment. Sustained exposure to inflammatory stimuli and oxidation induced by uric acid may also lead to the formation of local immunosuppression in the liver, providing a relatively tolerant liver microenvironment that allows the survival and growth of foreign tumor cells 87.

By inducing oxidative stress in both hepatocytes and pancreatic β, hyperuricemia can result in the development of insulin resistance and growth inhibition, which is associated with ectopic lipid deposition in the liver 88, 89. And a study showed that increased SUA levels were associated with hypoadiponectinemia, while the lack of adiponectin promoted the progression of hepatic steatosis, fibrosis, and hepatic tumor formation 90. Alternatively, uric acid can originate from fructose metabolism and when fructose is metabolized, there is a transient decrease in ATP levels, which induces oxidative stress and mitochondrial dysfunction, playing a key role in hepatic steatosis 91, 92. Because liver steatosis is linked with liver cell injury and inflammation, inflammatory cells such as neutrophils may be recruited during NAFLD, providing a fertile microenvironment for metastasis 79, 87. MMP13, one of the MMPs, is capable of cleaving various components of the ECM, and adhesion proteins are found to be significantly upregulated in NAFLD and contribute to tumor cell extravasation and establishment of metastases in the liver microenvironment 78. In addition, elevated circulating insulin-like growth factor (IGF-1) levels produced from a fatty liver promote liver metastasis not only through a direct paracrine effect on tumor cell survival and proliferation but also through indirect effects involving the host microenvironment and proinflammatory responses 93.

It's also worth mentioning that in non-small cell lung cancer patients who had SUA levels over 7.49mg/dL, the most common organ of metastasis was the brain 31, but the mechanism remains unclear. And the role of hyperuricemia as an independent risk factor for the initiation and progression of cancer is actually still controversial, which it may differ according to sex. It has been suggested that an increased SUA level might be a valuable long-term surrogate marker rather than an independent risk factor or even a carcinogenic substance itself, because increased SUA is also indicative of a lifestyle at increased risk for cancer 94. A cohort study in Taiwan has reported that uric acid protected against the development of cancer, and it showed that low serum uric acid levels were associated with a higher risk of all cancer mortalities relative to high serum uric acid levels 95, 96. In addition, a trend toward a negative association between gout and breast cancer has been reported, while there was a higher risk for male gout patients to develop prostate cancer, and this sex difference in the prevalence of hyperuricemia was correlated with the activity of sex hormones 36. As a result, additional studies with higher quality are needed to provide a precise determination of the relationship between high SUA and cancer development, particularly with regard to the sex and specific sites of malignancies.

In summary, this review examines the novel idea that uric acid may be an important risk factor for cancer when humans develop a high concentration of SUA. Hyperuricemia may also contribute to the metastasis of some cancers, but the precise mechanism still needs further exploration. We also suggest a new target that may integrate inflammation, oxidative stress, and cell cycle arrest, which have been largely neglected, but are known to be responsive to drug treatment. Based on preliminary clinical evidence, we suggest that drugs that lower serum uric acid might be useful to slow or delay the progression of cancer development. But the specific or approximate extent to which lowering uric acid remains unclear, and recently a cohort study has suggested that an SUA level of 5.7 mg/dL (6mg/dL in males and 4mg/dL in females) is considered safe with respect to mortality 97. Repurposing these existing drugs may therefore be a novel strategy for management of some refractory cancers, but the application of those drugs needs further analyses.

## Figures and Tables

**Figure 1:**
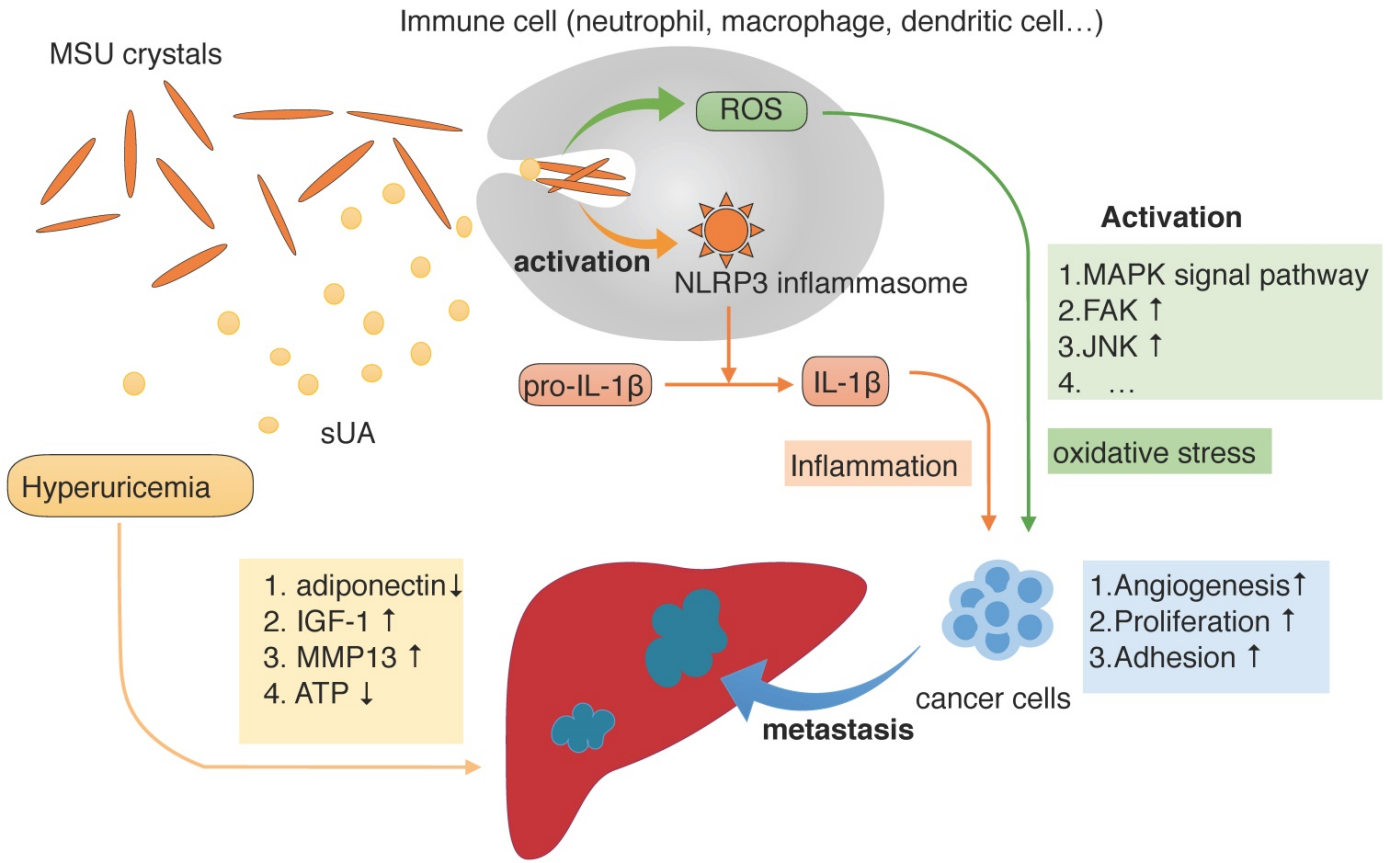
** The progression of cancer and metastasis to the liver during hyperuricemia conditions.** During hyperuricemia, the main forms of uric acid are soluble uric acid (sUA) and monosodium urate (MSU) crystals. As one of the damage-associated molecular patterns that alert the immune system to injurious situations, MSU crystals are detected and ingested by phagocytes, which stimulate the NOD-like receptor family and pyrin domain containing 3 (NLRP3) inflammasomes and generate reactive oxygen species (ROS) mainly in intracellular way. NLRP3 inflammasomes then promote cleavage of IL-1β into its active form, thus producing the IL-1β proinflammatory cytokine. Depending on the presence of MSU, sUA also participates in this process. The presence of ROS and IL-1β result in oxidative stress and inflammatory responses, respectively, which promote cancer development. Furthermore, hyperuricemia also alters the microenvironment of the liver, making it more likely that cancer will metastasize.

**Table 1:** The mediators of the development of cancer in the presence of high serum uric acid levels

Cause	Factor	Cancer development
activated NLRP3 inflammasome	Neutrophils, macrophages	Cancer risk ↑
chronic low-grade inflammation	CCL2 → monocytes	
VEGF, CDK inhibitor, MMPs	ROS ↑	
MMPs, EMK, FAK	ROS ↑	Liver metastasis risk ↑
NAFLD	Insulin resistance, hypoadiponectinemia, fructose metabolism	

## Abbreviations

CCL2: chemokine (C-C motif) ligand 2
CRC: colorectal cancer
DAMPs: damage-associated molecular patterns
ECM: extracellular matrix
FAK: focal adhesion kinase
EMT: epithelial-mesenchymal transition
MAPK: mitogen activated-protein kinase
Mets: metabolic syndrome
MMPs: matrix metalloproteinases
MSU: monosodium urate
IGF-1: insulin-like growth factor
IL-1Ra: IL-1 receptor antagonist
JNK: Jun N-terminal kinase
NAFLD: non-alcoholic fatty liver disease
NASH: non-alcoholic steatohepatitis
OS: overall survival
RET: rearrangement during transection
RFS: recurrence free survival
ROS: reactive oxygen species
RPTKs: receptor tyrosine kinases
SUA: serum uric acid
sUA: soluble uric acid
XOR: xanthine oxidoreductase
VEGF: vascular endothelial growth factor
